# Removal of MS2 and fr Bacteriophages Using MgAl_2_O_4_-Modified, Al_2_O_3_-Stabilized Porous Ceramic Granules for Drinking Water Treatment

**DOI:** 10.3390/membranes12050471

**Published:** 2022-04-27

**Authors:** Nur Sena Yüzbasi, Paweł A. Krawczyk, Kamila W. Domagała, Alexander Englert, Michael Burkhardt, Michael Stuer, Thomas Graule

**Affiliations:** 1 Laboratory for High Performance Ceramics, Empa, Swiss Federal Laboratories for Materials Science and Technology, 8600 Dübendorf, Switzerland; pawel.krawczyk171@gmail.com (P.A.K.); domagalakamila11@gmail.com (K.W.D.); michael.stuer@empa.ch (M.S.); thomas.graule@empa.ch (T.G.); 2 Faculty of Materials Science and Ceramics, AGH University of Science and Technology, 30-059 Krakow, Poland; 3 Department of Water and Wastewater Treatment, Institute of Environmental and Process Engineering, Eastern Switzerland University of Applied Sciences, 8640 Rapperswil, Switzerland; alexander.englert@ost.ch (A.E.); michael.burkhardt@ost.ch (M.B.)

**Keywords:** drinking water, virus removal, MS2 bacteriophage, fr bacteriophage, granules, ceramic filters

## Abstract

Point-of-use ceramic filters are one of the strategies to address problems associated with waterborne diseases to remove harmful microorganisms in water sources prior to its consumption. In this study, development of adsorption-based ceramic depth filters composed of alumina platelets was achieved using spray granulation (calcined at 800 °C). Their virus retention performance was assessed using cartridges containing granular material (4 g) with two virus surrogates: MS2 and fr bacteriophages. Both materials showed complete removal, with a 7 log_10_ reduction value (LRV) of MS2 up to 1 L. MgAl_2_O_4_-modified Al_2_O_3_ granules possessed a higher MS2 retention capacity, contrary to the shortcomings of retention limits in pure Al_2_O_3_ granules. No significant decline in the retention of fr occurred during filtration tests up to 2 L. The phase composition and morphology of the materials were preserved during filtration, with no magnesium or aluminum leakage during filtration, as confirmed by X-ray diffractograms, electron micrographs, and inductively coupled plasma-optical emission spectrometry. The proposed MgAl_2_O_4_-modified Al_2_O_3_ granular ceramic filter materials offer high virus retention, achieving the criterion for virus filtration as required by the World Health Organization (LRV ≥ 4). Owing to their high thermal and chemical stability, the developed materials are thus suitable for thermal and chemical-free regeneration treatments.

## 1. Introduction

Safe and readily available drinking water is one of the major requirements of a healthy life. In 2017, 2.2 billion people had no access to safe drinking water, despite significant associated health risks [1]. Waterborne diseases can be readily transmitted through bacteria (e.g., *Vibrio cholerae*, *Legionella pneumophilia*, *Salmonella typhi*) and viruses (e.g., poliovirus, rotaviruses A–F, hepatitis A virus) and cause severe illnesses and deaths of millions of people [2].

Access to clean drinking water has been achieved based on several chemical, physical, and mechanical processes (such as heat treatment, chlorination, ozonation, chemical precipitation, or coagulation and flocculation and photochemical inactivation with UV irradiation) and filtration technologies, which have been proven to effectively remove or inactivate viruses or other microorganisms [3,4,5]. Application of some of these processes can sometimes be highly challenging in developing countries or rural areas in developed countries due to the high costs of treatment and distribution systems and a lack of or limited infrastructure [6,7].

One of the main strategies to address problems associated with waterborne diseases worldwide is to apply on-site water treatment systems, i.e., point-of-use (POU) or household water treatment (HWT) technologies, to reduce harmful microorganisms in water sources prior to consumption. Porous ceramic filters are widespread and increasingly used as drinking water treatment technologies [8,9,10,11], particularly in rural areas and developing countries [10,12].

Ceramic filters are highly advantageous as they are compatible with regeneration processes such as steam sterilization, calcination, backflushing, or chemical agents [2,13,14]. Such filters are generally effective in the removal of pathogens in the microporous range, such as bacteria and protozoa. This approach, however, fails in virus filtration due to rapid fouling of the nanometric pores (to trap viruses with typical dimensions of <100 nm), among other considerations [15,16,17]. The application of filters with nanopores may be limited due to the high pressure drop (high energy consumption), their low throughput, and especially the risk of fast blocking by colloidal fouling.

Viruses are nano-sized amphoteric microbes with a varying surface charge depending on the individual virus type and strain [12,18,19,20]. The net surface charge of viruses is pH- and surface chemistry-dependent [12,19,21,22]. An increase in the pH of the medium can lead to an increase in the ionization of carboxyl and sulfhydryl groups, and a decrease in ionization of amine groups at the surface of viruses [12,17]. Typically, isoelectric points (IEP) of viruses vary between pH 3 to 9, leading to the presence of both positively and negatively charged viruses in natural waters, depending on the virus type [17,20,23]. Surface characteristics can play a significant role in virus removal/inactivation in porous media [9,20,21,24]. Attempts have been made to predict the adsorption characteristics of viruses in porous media using the DLVO theory to model the electrostatic and van der Waals forces [12,17,21]. Additionally, previous studies have demonstrated the effect of non-DLVO factors on virus-media sorption and/or inactivation, such as hydrophobicity [23,25,26] effects arising from structural incompatibility between viruses and sorbents [20,24], roughness of the deposition surface/sorbent [27], and water chemistry [28,29].

Metal oxide surfaces are expected to possess a positive surface charge at pH values below the isoelectric point [12,30,31], which can in turn promote the attraction of viruses. In fact, functionalization of conventional depth filter surfaces, e.g., sand filters or diatomaceous earth or fiber structures, by metal oxides such as iron oxide [32,33,34,35], aluminum oxide [15,33,36], copper oxide [15,35,37], magnesium oxide [38], hydrated oxides of yttrium [29], and zirconium [2,29], resulted in enhanced virus retention.

In addition to the metal oxides mentioned above, MgAl_2_O_4_ is also known for its high IEP (~pH = 11.8) [39,40]. Even if Al_2_O_3_ or MgO were previously studied for virus retention applications, to the best of our knowledge, MgAl_2_O_4_ has not been implemented as an adsorbent for virus removal. Utilization of MgAl_2_O_4_ for water filtration applications was only performed by Kamato et al., for the removal of submicron-sized colloidal particles (simulating bacteria) from a suspension [41]. MgAl_2_O_4_ can be a suitable material for ceramic filters due to its non-toxicity, low cost, and excellent chemical stability. The latter property enables easy regeneration of the filter materials by backflushing [8,36], thermal [42], acidic, or basic treatment [43], without any phase transition. Such phase changes were previously observed in Cu_x_O_y_-based granules upon thermal treatment, which makes these materials less functional for potential applications in water filtration [15].

To this end, this study investigates the development of granular ceramic filter materials through the modification of Al_2_O_3_ granules with MgAl_2_O_4_ nanoparticles (Mg-NP), where the granular structures were developed by the spray granulation technique. The granules were calcined at 800 °C for further consolidation. The granular materials were tested in flow tests to determine the retention capacity of two different bacteriophages (MS2 and fr bacteriophages), serving as surrogates for human pathogenic waterborne viruses. MS2 bacteriophage is often used and was chosen as a surrogate for apolar and negatively charged human enteric viruses [16,44], while fr bacteriophage was selected due its electropositive surface in water at pH in the range from 3 to 9 [45,46].

## 2. Materials and Methods

Materials. The synthesis of filter materials was achieved using the spray granulation technique. Commercially available MgAl_2_O_4_ nanoparticles (d_v50_ = 0.2–0.3 µm, spinel S25CR, Baikowski SA, France, purity ≥ 99%, surface area of 21–24 m^2^∙g^−1^) and plate-like Al_2_O_3_ (d_v50_ = 6–12 µm, white sapphire alumina, Merck Group, Germany, purity > 99.0%, surface area of 1–2 m^2^∙g^−1^) were selected as starting materials. PAA5 (Polyacrylic acid, 50% soln. in water (MW ~ 5000), Polyscience, Inc., Warrington, PA, USA) and polyvinyl alcohol (PVA, MW 31,000–50,000, 98–99% hydrolyzed, Sigma Aldrich, St. Louis, MO, USA) were used as a dispersant and a binder, respectively.

Spray granulation. Materials were developed using the Büchi Mini Spray Dryer B290 (Büchi Labortechnik AG, Flawil, Switzerland) [39,40]. The details regarding slurry preparation and synthesis parameters are provided in the Supplementary Materials. To describe the materials, the following nomenclature is used throughout this paper: MgAl, Al, Al-Pl, Mg-NP, for MgAl_2_O_4_-modified Al_2_O_3_ granules, Al_2_O_3_ granules, plate-like Al_2_O_3_ powder (white sapphire), and MgAl_2_O_4_ nanoparticles (spinel S25CR), respectively, and summarized in Table 1. In order to consolidate the granules, remove the polymer binder matrix, and achieve strong bonding between Mg-NP and Al-Pl, granules were calcined in air at 800 °C with a heating (and cooling) rate of 5 °C·min^−1^ and a 1 h dwell time in PY 12 H (Pyrotec Brennofenbau GmbH, Osnabrück, Germany).

Characterization. Synthesized materials were characterized using X-ray diffraction (XRD), N_2_ physisorption, energy-dispersive X-ray spectroscopy (EDX), zeta potential, laser diffraction (LD), and helium pycnometry. The details of the characterization measurements are provided in the Supplementary Materials.

Bacteriophages and filtration tests. Two different bacteriophages, *Escherichia* phage MS2 (MS2; diameter = 25 nm, DSMZ 13767, Braunschweig Germany, IEP ~3.5–3.9 [17]) and *Escherichia coli* bacteriophage fr (fr, diameter = 19 nm, ATCC 15767-B1, Virginia, USA, IEP ~8.9–9.0 [45,46]), were used as virus surrogates. The associated host organisms for MS2 and fr were *Escherichia coli* strain W1485 (DSM-5695, Braunschweig, Germany) and *Escherichia coli* strain 3300-141 (ATCC 19853, Manassas, VA, USA), respectively.

Virus solutions with a concentration of 10^8^ PFU∙mL^−1^ in TRIS buffer (0.02 M tris(hydroxymethyl)-aminomethane (Merck Group, Darmstadt, Germany) and 5 mM magnesium sulfate (Merck Group, Darmstadt, Germany), pH = 7.3) and their host bacteria were purchased from the Culture Collection of Switzerland (CCOS, Wädenswil, Switzerland). For enumeration of phages, the double agar layer (DAL) method was applied according to the US EPA Method 1602, 2001 [47] (as described in the Supplementary Materials in detail). The phage concentration (I_f_) was calculated, accounting for the dilution (D), using the following Equation (1):

(1) $$I_{f}=\mathrm{Number}\;\mathrm{of}\;\mathrm{plaques}\cdot D\;[\mathrm{PFU}\;{\mathrm{mL}}^{-1}]$$

Additionally, log_10_ virus removal (LVR) efficiency was determined for each tested material based on Equation (2), where I_0_ is the initial phage concentration:

(2) $$LVR=log\frac{I_{0}}{I_{f}}\;$$

Virus retention tests were performed in a laboratory-scale filtration setup (Figure S1), where 4 g of granular material was placed over glass fiber filter paper (pore size 0.4 μm, binder free, Macherey-Nagel filters) in a 70 mm-long cartridge with a diameter of 15 mm. The flow rate of the solution was adjusted to 300 mL∙h^−1^ and the pressure inside the cartridge at the beginning of the tests was measured as 0.4 bars.

To assess the virus retention of the materials, continuous-flow filtration tests were conducted using MS2 or fr bacteriophages, where the initial concentration of the virus solutions was fixed to 10^7^ PFU∙mL^−1^ in TRIS buffer. The filtration characteristics of the developed granular ceramic filter materials were assessed based on dead-end filtration tests. Approximately 20 mL of permeate was collected after 250, 650, 1000, 1250, 1650, and 2000 mL of virus solution passed through the filtration medium to follow the decline in the LRV and thus the saturation trend of the granular ceramic filter materials. For each material, two cartridges containing 4 g of granular ceramic filter material were prepared to verify the reproducibility of the results.

Furthermore, to assess the solubility of the materials in contact with the filtration medium, magnesium and aluminum content of permeates were measured by inductively coupled optical emission spectrometry (ICP-OES, Acros, Spectro Analytical Instruments GmbH, Kleve, Germany).

## 3. Results and Discussion

### 3.1. Characterization of Starting Materials

**Figure 1:**
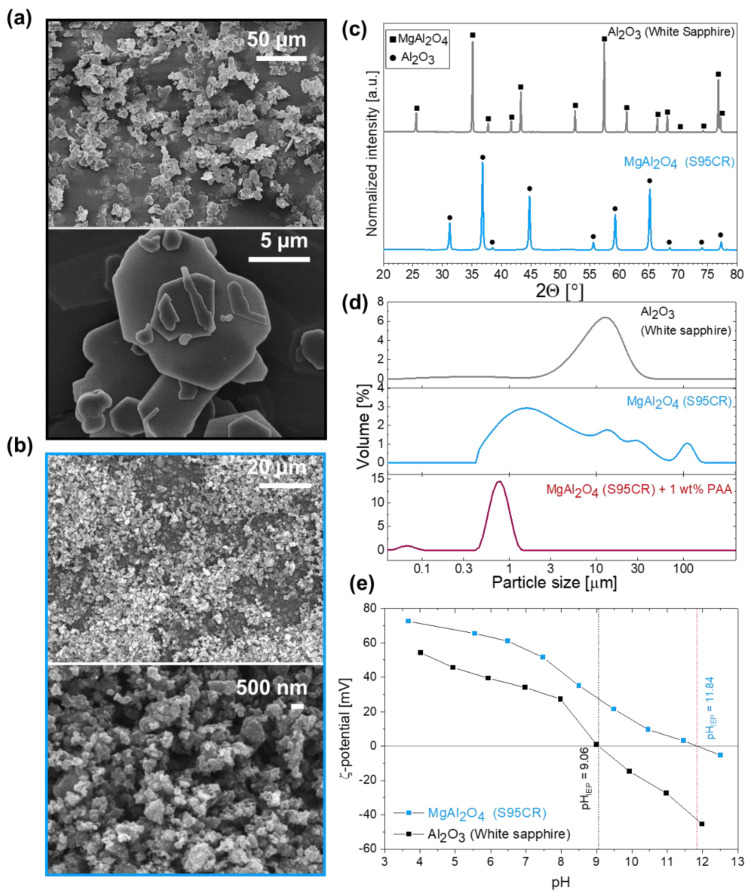
Characterization of the starting materials, MgAl_2_O_4_ nanoparticles and Al_2_O_3_ (white sapphire), as represented by blue and gray colors, respectively. Electron micrographs of (**a**) Al_2_O_3_ (white sapphire, Al-Pl) and (**b**) MgAl_2_O_4_ nanoparticles (Mg-NP), (**c**) X-ray diffractogram, (**d**) particle size distribution, and (**e**) zeta potential as a function of pH.

Figure 1 shows the surface morphology of the raw powders. Al-Pl (Figure 1a) is composed of micron-sized plate-like particles with various irregular shapes, which tend to pile on each (i.e., agglomeration). Similarly, Mg-NP (Figure 1b) possesses a rough surface texture, as a result of irregularly shaped nanoparticle aggregate formation. The specific surface area (SSA) of raw materials was determined as 1.7 and 24.6 m^2^·g^−1^ (Table 2), for Al-Pl and Mg-NP, respectively.

Crystalline phases of the commercial raw powders were determined using XRD and are shown in Figure 1c. The powders were crystalline without the presence of impurity phases. The diffraction peaks of Al-Pl are characteristic of trigonal α-Al_2_O_3_ with a rhombohedral (corundum) structure and *R*-3 *c* (167) space group (PDF: 43-1484). The diffraction pattern of Mg-NP corresponds to that of cubic MgAl_2_O_4_ with a spinel structure and *F d* -3 *m* (227) space group (PDF: 21-1152).

The particle size distribution of the starting materials is shown in Figure 1b, and d_v10_, d_v50_, and d_v90_ values are summarized in Table 2. The volume-based LD measurements for alumina platelets represented a monomodal and relatively broad particle size distribution. d_v10_ of 1 µm, d_v50_ of 12 µm, and d_v90_ of 26 µm were determined for alumina platelets, in agreement with the company-provided values. Note that the shape of particles has a strong impact on the particle size measurements, since the particle size distribution of non-spherical particles is calculated on the basis of equivalent spherical diameters [48,49].

On the other hand, spinel nanoparticles showed a broad and polymodal particle size distribution with a d_v50_ of 2.7 µm, in disagreement with the company-provided values (0.2–0.3 µm). The volume-based LD measurements thus indicate strong agglomeration of spinel nanoparticles, as seen on the electron micrographs, requiring extensive milling to re-disperse Mg-NP prior to granulation. Therefore, PAA5 was selected as a dispersant to stabilize Mg-NP. The influence of dispersant concentration and milling time on particle size distribution was critically assessed and further explained in the Supplementary Materials (Figure S2). The addition of 1 wt.% dispersant and 10 h of milling allowed to reduce the particle size distribution of Mg-NP (d_v10_ of 0.6 µm, d_v50_ of 0.8 µm, and d_v90_ of 1.1 µm), as shown in Figure 1b.

The zeta potential of alumina platelets and spinel nanoparticles was measured as a function of pH, as represented in Figure 1c. Both starting materials showed a positive zeta potential (above 30 mV) in the pH range typical for drinking water (pH 6 to 8). The IEP of AL-Pl and Mg-NP was determined at 9.06 and 11.84, respectively (Table 2), in agreement with the literature [39,40,50].

### 3.2. Granulation

Spray-drying allows changing the granule morphology by tuning the slurry properties and granulation parameters. Preliminary tests pre-established an appropriate solid load of the dispersion, nozzle type, and binder fraction to optimize the granulation yield, material size, porosity, and surface area. Only a marginal difference in particle size distribution and morphology of the granules could be observed depending on the nozzle type, i.e., ultrasonic or two-fluid nozzle (Figure S3). Due to the ease of handling, the two-fluid nozzle was selected for further experiments. On the contrary, the binder content had a distinct impact on the granule morphology and size, as represented in Figure S5. The PVA content was varied between 0 and 5 wt.% (referring to the total amount of all powders in the slurry). At least 2 wt.% of binder was found to be required to form well-defined granules rather than a mix of broken granules and loose powder. Higher amounts of binder (5 wt.% PVA), however, caused the formation of large granule agglomerates and thus heterogeneous, bimodal size distributions with diameters up to 1 mm. The optimal fraction of PVA was thus found to be 2 wt.% (6.2 vol.%) in order to obtain homogenous, spherical-shaped granular material. Finally, high SSA, as one of the important prerequisites for successful virus adsorption, could be achieved by lowering the solid loading within the powder slurry for spray granulation. Lowering the solid load from 20 to 10 vol.% almost doubled the SSA of MgAl granules from 4.0 to 7.6 m^2^∙g^−1^.

**Figure 2:**
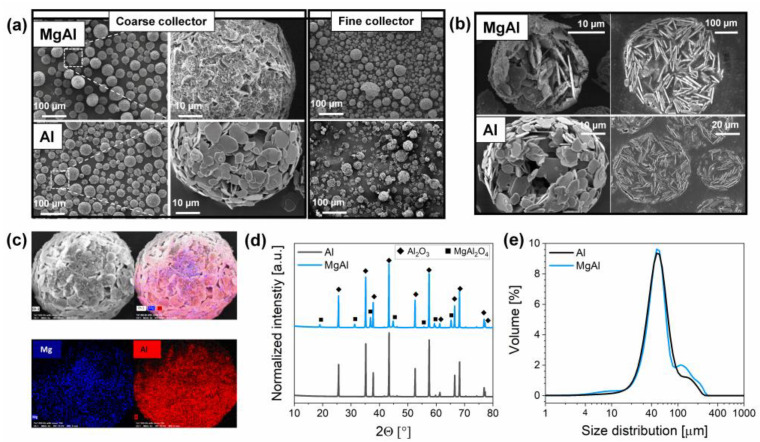
Characterization of the spray-dried Al (―) and MgAl (―) granules that were calcined at 800 °C. (**a**) Electron micrographs, (**b**) sub-surface morphology of the broken (left) or polished (right) granules, (**c**) elemental mapping of MgAl granules, (**d**) X-ray diffractograms, and (**e**) particle size distribution of the granules.

Figure 2a represents the surface morphology of the MgAl and Al granules with a solid loading of 10 vol.% in the presence of 2 wt.% PVA (and 1 wt.% PAA5 in the case of MgAl for spinel dispersion) after their calcination at 800 °C. Electron micrographs reveal the presence of spherical granules in both materials, which have been collected in the coarse collector of the spray-dryer (as illustrated in Figure S4). However, observing the granules collected in the fine collector of the spray-dryer revealed that they had been broken and that the granulation was especially poor in the case of Al compared to MgAl. This can be attributed to an additional binding effect from MgAl_2_O_4_ nanoparticles that tend to adhere strongly to each other, as previously described by Kendall et al. [51,52]. SEM images of the broken or polished granules in Figure 2b display the sub-surface morphology of the materials. Both materials possessed a highly porous internal structure (as previously also confirmed by mercury intrusion porosimetry [15]), a critical feature to ensure effective water flow during filtration, as a result of randomly oriented alumina platelets and the low solid load of the ceramic slurry. To evaluate the compositional homogeneity between Al_2_O_3_ and MgAl_2_O_4_, EDX measurements were carried out. The elemental maps of aluminum and magnesium provided in Figure 2c revealed that Mg-NP were homogeneously distributed on the alumina platelets and within the granule volume (surface and sub-surface), as also confirmed in Figure S6. There was no phase change during granulation and calcination steps, as confirmed by the diffraction patterns of Al and MgAl in Figure 2d that show the presence of α-Al_2_O_3_ and cubic MgAl_2_O_4_.

The particle size distribution of the granules (Figure 2e and Table 2) was affected only marginally by the presence of Mg-NP. On the contrary, the introduction of Mg-NP resulted in a four times larger specific surface area for MgAl (7.6 m^2^∙g^−1^) granules compared to Al granules (1.7 m^2^∙g^−1^) when alumina was partially substituted by a material with a larger specific surface area, such as Mg-NP (24.6 m^2^∙g^−1^). The porous structure of the granules was further characterized by using mercury intrusion porosimetry (MIP). The cumulative pore volume porosity and pore size distribution of the granules are represented in Figure S7. The cumulative pore volume of Al and MgAl was only marginally different, detected as 1.01 and 0.91 cm^3^/g, respectively, indicating that the presence of Mg-NP did not block the pores of the granules. In both materials, two distinct pore size ranges were noticeable between 0.5 to 2 μm and 8 to 20 μm.

### 3.3. Filtration Tests and Virus Removal Performance

**Figure 3:**
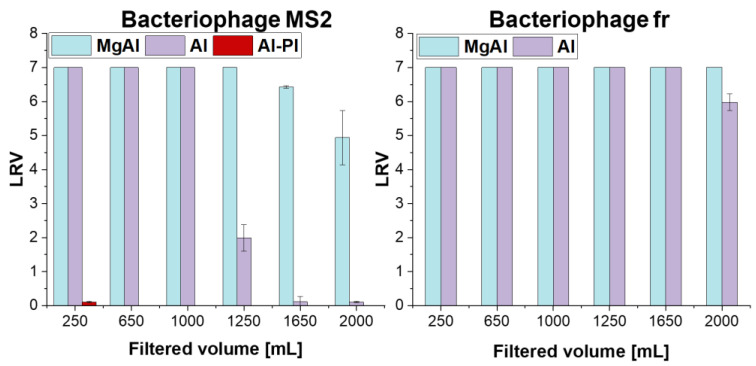
Retention performance of the granules based on MS2 and fr log_10_ removal as a function of filtered volume. All filter media contained the same amount of material (4 g).

Prior to the tests with the granular material, control tests (with cartridges containing only glass fiber filter paper) were performed to examine bacteriophage binding on the filter paper. These tests confirmed that the presence of glass fiber filter paper did not have any contribution in the removal of bacteriophages. LRV results obtained during dead-end filtration tests performed using 4 g of ceramic filter materials are presented in Figure 3. Additionally, LRV was also plotted against normalized filtrate volume per filter bed volume, where bed volumes were calculated as 4.8 and 6.2 mL for granules and Al-Pl, respectively, as shown in Figure S8. Figure 3 shows that the MS2 phages can be effectively retained and completely removed from water up to 1 L with 4 g of both synthesized granules. The granular structure, in a similar context with depth filtration, enabled higher contact time between the adsorbent and MS2-contaminated water, which permitted effective physisorption and chemisorption of the contaminants, when compared to surface filtration [53]. However, there was a sharp decline in LRV of Al granules after 1.25 L, and ultimately no virus removal after 1.65 L, indicating that saturation of granules was reached. On the other hand, virus retention of MgAl slightly decreased after 1.65 L from LRV 7 to 5, while still meeting the WHO standards for drinking water (LRV ≥ 4) over 2 L [54].

As a point of comparison, non-granulated alumina platelets showed significantly poorer virus retention capacity and there was no removal of negatively charged MS2 bacteriophages (Figure 3). Due to the high powder packing density, filtration with Al-Pl was significantly more challenging compared to filtration with the granules and accompanied by a severe pressure drop in the cartridges. This clearly illustrates the importance of the microstructure (e.g., tortuosity) and porosity of the filter media during filtration. Due to the filtration challenges in Al-Pl filter media, they were only evaluated up to 250 mL, and the tests continued thereafter with granular materials only. Filtration tests revealed that despite a high positive surface charge of Al-Pl (IEP = 9.1), there was no removal of negatively charged MS2 bacteriophages (IEP ≈ 3.5 [17]) at pH 7.3. Such a high charge difference between the filter and bacteriophage surface has been reported to lead to a virion sorption through electrostatic forces [20,38,55]; however, it is insufficient in explaining our experimental data. Indeed, despite attractive electrostatic forces, the poor retention performance of Al-Pl may be a result of: (i) a significantly low surface area due to platelet agglomeration via the basal planes, and thus a small number of adsorption sites in Al-Pl, and/or (ii) the formation of preferential flow paths, e.g., short-circuits of the filter. The latter leads to an insufficient contact of contaminated water with the filter surface for adsorption of bacteriophages, quickly saturating the little-exposed filter surface area on the flow paths. The former prevents the fulfilment of a key prerequisite for effective virus retention: having a high number of adsorption sites. Indeed, a higher virus removal capacity is often not solely correlated with the IEP of viruses and sorption surface, but also with the surface area and roughness of the sorption media [27]. Dika et al. demonstrated that substrate roughness has an impact on the adhesion of bacteriophages, where weaker adhesion was observed on a low-roughness surface (glass) when compared to substrates with a higher roughness (polypropylene or stainless steel). Our results strongly suggest that the slower saturation of MgAl granules observed in filtration tests with MS2 bacteriophage can be linked to the larger surface area, surface roughness, and higher adsorption sites for viruses provided by spinel nanoparticles, when compared to Al granules.

One important characteristic of a virus is its IEP, which represents the pH value at which the surface charge of a virus is zero. Usual IEPs of viruses range from 3 to 7 [17]. To cover the IEPs of relevant viruses found in water in a wider range and have a better understanding on the potential contribution of electrostatic forces, MS2 filtration tests were complemented by separate filtration tests using fr bacteriophages. The filtration tests with fr bacteriophages revealed successful retention of up to 2 L of contaminated water for Al and MgAl granules. There was a small decline only in LRV of Al granules from LRV 7 to 6, after 1.65 L, while MgAl granules could successfully achieve complete removal of fr bacteriophages even after 2 L.

fr bacteriophage has a high IEP of 8.9 according to literature-reported values [56,57,58]. The filtration tests with fr bacteriophages revealed successful retention up to 2 L of contaminated water, in spite of the low zeta-potential difference between the filter material and virus surrogate and the resulting low electrostatic interaction forces’ contributions. A more detailed literature review, however, shows that the reported IEP of fr bacteriophage varies widely, from 3.5 to 9.0 [55]. Recent studies experimentally validated by light scattering and electrophoretic mobility measurements show that fr bacteriophages have mostly a negative surface charge [20,21,55]. Armanious et al. theoretically estimated the surface charge of fr bacteriophage based on the ionizable amino-acids and the tertiary structure of fr capsid protein and reported its surface charge and IEP as −2.5 × 10^2^ C·m^−2^ and 4.5, respectively [20]. Due to the contradictory findings in the literature with respect to the surface charge of fr bacteriophages, the present retention results need to be assessed considering two scenarios: fr bacteriophages have a (i) negative surface charge and (ii) positive surface charge. In the first scenario, short-range attraction forces, i.e., van der Waals forces or hydrophobic effects, may dominate or replace the electrostatic forces in virion sorption. Following the second scenario, fr adsorption may occur similarly to MS2, driven by longer range electrostatic forces which can be further complemented with attractive van der Waals forces and hydrophobic effects. In the latter scenario, the retention performance difference obtained in filtration with fr bacteriophages and MS2 bacteriophages can be linked to the level of hydrophobicity caused by differences in the surface polarities. Armanious et al. calculated the relative hydrophobicity of MS2 and fr bacteriophages and suggested that fr bacteriophage experienced larger contributions from the hydrophobic effect due to its higher apolarity [20].

Ongoing efforts clearly demonstrate the complexity of the interactions that play a critical role in the virus trapping, and further investigations are necessary to validate the exact mechanisms as well as exclude or quantify additional contributions that may result from filter aging. According to the WHO International Scheme to Evaluate Household Water Treatment Technologies report [54], a typical virus concentration of challenge water and a minimum test water volume is indicated as 10^5^ PFU∙mL^−1^ and 20 L∙day^−1^ for laboratory verification tests of POU water filtration technologies, e.g., granular media and porous or membrane filters [59]. Test waters used in this study were spiked two orders of magnitude higher than suggested challenge concentrations (in the absence of humic acids or natural organic materials). Filtration tests with 2 L of MS2- or fr-contaminated water in controlled systems show outstanding adsorption properties of MgAl granules (with 4 g) and bring the material system closer to testing in real water systems. These materials need to be tested for longer periods with higher volumes of challenge test waters based on guideline values of the WHO [54,60], prior to the application stage.

### 3.4. Characterization of Materials after Filtration

**Figure 4:**
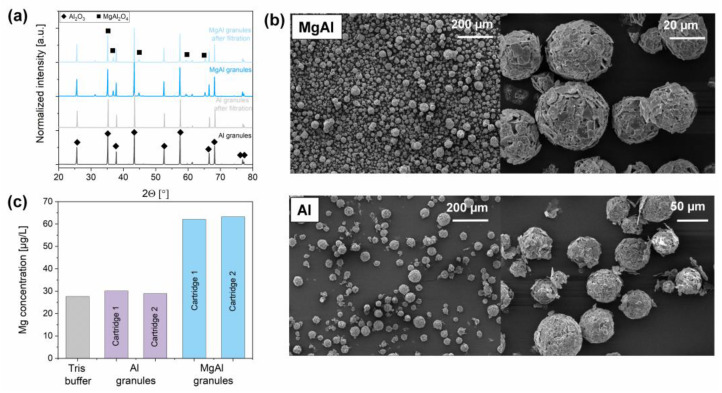
Characterization of the granules after filtration. (**a**) X-ray diffractograms, (**b**) electron micrographs, and (**c**) associated magnesium concentrations detected in permeate, as determined by ICP-OES.

Both materials were characterized following filtration in order to investigate the influence of water exposure on the morphology and phase composition of the granules. The phase composition of the granules was preserved during filtration according to X-ray diffractograms, as shown in Figure 4a.

The electron micrographs presented in Figure 4b indicate that the granular structure was preserved after their exposure to 2 L of contaminated water. Further tests on the stability of the granules were performed using ICP-OES to evaluate the dissolution of alumina and magnesium aluminate spinel to the form of Al^3+^ and Mg^2+^ cations in the permeate after filtration tests with fr bacteriophage. Aluminum was not detected after 2 L of filtration (where the detection limit of Al is 50 µg∙L^−1^), and only a small quantity of magnesium release (<60 µg∙L^−1^) was observed in the case of MgAl granules (Figure 4c). The magnesium concentration in the permeate, which is known to cause hardness in drinking water, was three orders of magnitude lower than the taste threshold value recommended by the WHO for drinking water (<25–50 mg∙L^−1^) [59].

As the limited lifetime is a severe technical challenge within an adsorption-based, dead-end filtration process, the thermal regeneration will be a beneficial alternative even after the filter itself starts losing its efficiency due to clogging of its pores and the occupation of all adsorption sites by the virus contaminants (and by concomitant humic acid and other competitive adsorbing water contaminants in real water systems). One of the main advantages of granular ceramic filter materials is the possibility of regenerating the filter media by thermal means [11,29]. Preliminary tests revealed that after heat treatment of the saturated MgAl filter at 400 °C in air, the virus retention capacity was recovered (Figure S9). However, it is important to mention here that the filters were not fully saturated and filtration after regeneration was performed with only 250 mL of MS2 solution (10^7^ PFU /mL). Therefore, optimized regeneration conditions need be developed by assessing the process parameters such as temperature and regeneration cycles in the presence of competitive adsorbing water contaminants, as envisaged to be conducted in the future.

## 4. Conclusions

In this work, we shed light on the structure–performance relationship of spray-dried granules as a ceramic filter material for virus removal in drinking water applications. The following conclusions were reached under the observations of this study:

1. The presence of homogenously distributed Mg-NP in Al_2_O_3_ granules offers effective means to enhance adsorption sites of virus surrogates (MS2 and fr bacteriophages).
2. MgAl_2_O_4_-modified Al_2_O_3_ granules exceeded the retention performance of pristine Al_2_O_3_ granules, as revealed through flow tests.
3. MgAl_2_O_4_-modified Al_2_O_3_ granules possess promising adsorption properties, and could successfully achieve a log_10_ reduction of 5 and 7 of MS2 and fr bacteriophages, respectively, with 4 g of MgAl after 2 L of filtration.
4. There was no degradation in phase composition and morphology of the granules upon filtration.
5. No aluminum nor significant magnesium leakage was detected during the filtration, suggesting a high stability of the developed materials as a result of consolidation at 800 °C.
6. Preliminary regeneration tests indicated that the developed granular ceramic filter materials can be potentially reused after thermal treatment.

The underlying mechanism of successful virus retention is still not clear; however, experimental findings suggest that highly porous granular structures play a key role in the removal of bacteriophages. It is suggested that it enables a good permeability and thus contact between the material and the influent.

The current study therefore highlights the potential of MgAl_2_O_4_-Al_2_O_3_ granules for drinking water treatment. Prior to real applications, however, the filter materials developed in this study need to be tested with more complex water chemistries, such as the presence of complexing factors (e.g., natural organic matter, different pH) and regeneration options (e.g., by thermal means) need to be evaluated to increase the lifetime and reuse the absorber material.

## Figures and Tables

**Table 1:** Material nomenclature.

	Starting Materials	Spray-Dried Granules
Al-Pl	plate-like Al_2_O_3_ powder (white sapphire)	-
Mg-NP	MgAl_2_O_4_ nanoparticles (spinel S25CR)	-
MgAl	-	MgAl_2_O_4_-modified Al_2_O_3_ granules
Al	-	Al_2_O_3_ granules

**Table 2:** Particle size distribution (d_v90_, d_v50_, and d_v10_), surface area, density, and IEP of starting powders and synthesized granules.

	Starting Materials			Granules	
	Al-Pl	Mg-NP	Mg-NP/1 wt.% PAA	Al Granules	MgAl Granules
Particle size					
d_v90_ (µm)	25.7	31.7	1.1	98.20	123.5
d_v50_ (µm)	11.6	2.7	0.8	51.69	52.02
d_v10_ (µm)	1.1	0.5	0.6	28.20	26.37
Surface area (m^2^∙g^−1^)	1.7	24.6	-	1.7	7.6
Cumulative pore volume (cm^3^∙g^−1^)	-	-	-	1.01	0.91
Density (g∙cm^−3^)	3.94	3.80	-	-	-
IEP	9.06	11.84	-	-	-

## Data Availability

Not applicable.

## Supplementary Materials

The following supporting information can be downloaded at: https://www.mdpi.com/article/10.3390/membranes12050471/s1. Details regarding characterization techniques, spray-drying, and virus removal experimental set-up descriptions, additional material characterization and retention results (PDF). References [61,62,63,64,65] are cited in the supplementary materials.
