# Supplementary material for: Removal of MS2 and fr Bacteriophages Using MgAl2O4-Modified, Al2O3-Stabilized Porous Ceramic Granules for Drinking Water Treatment
Source: Membranes (Basel). 2022 Apr 27;12(5):471. doi: 10.3390/membranes12050471 (PMC9145336; doi:10.3390/membranes12050471)
Supplement: Supplementary file 1 [file membranes-12-00471-s001.zip › membranes-1673785-supplementary.pdf]

membranes

MDPI

Supplementary material

Removal of MS2 and fr Bacteriophages Using $MgAl_2O_4$-Modified, Al_2O_3-Stabilized Porous Ceramic Granules for Drinking Water Treatment

Nur Sena Yüzbasi [1,*], Paweł A. Krawczyk [1,2], Kamila W. Domagała [1,2], Alexander Englert [3], Michael Burkhardt [3], Michael Stuer [1] and Thomas Graule [1]

[1] Laboratory for High Performance Ceramics, Empa, Swiss Federal Laboratories for Materials Science and Technology, 8600, Dübendorf, Switzerland; pawel.krawczyk171@gmail.com (P.A.K.); domagalakamila11@gmail.com (K.W.D.); michael.stuer@empa.ch (M.S.); thomas.graule@empa.ch (T.G.)

[2] Faculty of Materials Science and Ceramics, AGH University of Science and Technology, 30-059 Krakow, Poland

[3] Department of Water and Wastewater Treatment, Institute of Environmental and Process Engineering, Eastern Switzerland University of Applied Sciences, 8640, Rapperswil, Switzerland; alexander.englert@ost.ch (A.E.); michael.burkhardt@ost.ch (M.B.)

* Correspondence: sena.yuezbasi@empa.ch

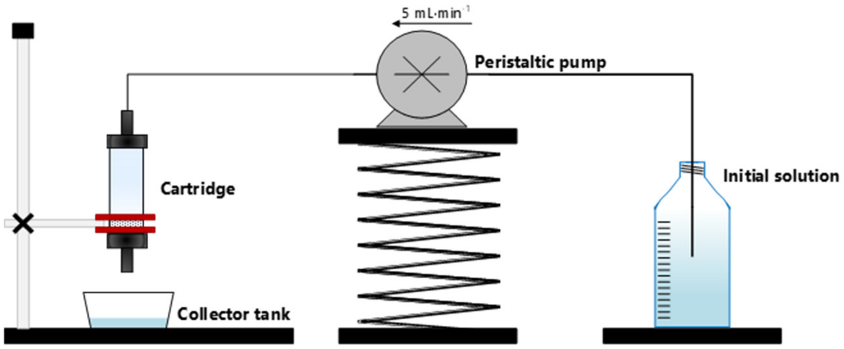

Figure S1. Schematic diagram of experimental set-up for virus removal tests.

Spray granulation. In the case of the synthesis of Al_2O_3 granules, Al_2O_3 platelets (white sapphire) were mixed with 3 mm zirconia milling balls (1:1 powder:balls weight ratio) in ultrapure water (MicroPure UV System, Thermo Scientific, Waltham, Massachusetts, USA) to obtain a slurry with a powder solid load of 10 vol.%. The resulting slurry was subjected to ball-milling on a rolling bench at 50 rpm for 24 h, and slurry preparation was followed by the addition of aqueous PVA solutions as a binder. The quantity of PVA was fixed at 2 wt.% with respect to the total amount of all powders in the slurry. PVA solutions were prepared through dissolution of PVA for 6–7 hours in water at ~90 °C. For the synthesis of $MgAl_2O_4$-modified Al_2O_3 granules, previously dispersed $MgAl_2O_4$ was added to alumina slurries to ensure a homogenous distribution of the nanoparticles on the Al_2O_3 matrix. In this context, prior to the addition of $MgAl_2O_4$, appropriate amounts of PAA5 and NH_3 (PAA5:NH_3 molar ratio = 1.5) were mixed in ultrapure water. Subsequently, all components were combined, and the slurry was subjected to 24 h of ball-milling (with the addition of 1 and 3 mm (1:1 ratio) zirconia milling balls in a 1:5 powder:balls weight ratio). The weight ratio between $MgAl_2O_4$ and Al_2O_3 was fixed to 0.15:0.85 in the final material. The quantity of PAA5 was selected based on the data derived from saturation point determination, as shown in Figure S1, and was fixed to 1 wt.% of the $MgAl_2O_4$ powder. After 24 hours of ball-milling, both slurries were mixed with a subsequent addition of PVA (2 wt.%) and left to mix for an additional 2 h.

All the spray-drying experiments were performed with a Büchi Mini Spray Dryer B290 (BÜCHI Labortechnik AG, Flawil, Switzerland) working in co-current mode, equipped with a cyclonic collector and Dehumidifier B296 using a two-fluid nozzle with a diameter of 0.7 mm. The process parameters were selected as follows: nozzle inlet temperature = 155 °C, aspiration rate 60%, air flow rate 20 $m^3 \cdot h^{-1}$, and feed rate 0.6 $mL \cdot min^{-1}$. Granules were collected in two collectors: a coarse collector at the bottom of the drying chamber for large granules and a fine collector after the cyclone separator for primary particles and small granules, as schematically illustrated in Figure S2.

Dispersant evaluation. The dispersant concentration in a slurry needs to be critically assessed, i.e., the polymer presence should be high enough to completely cover the particle surface to prevent particle close contacts or to counteract van der Waals forces, and low enough to avoid further flocculation of the non-adsorbed free polymer induced by depletion forces [1,2]. Therefore, the optimum PAA5 concentration for $MgAl_2O_4$ dispersion was examined based on zeta-potential measurements, as illustrated in Figure S1. The surface charge of $MgAl_2O_4$ sharply decreased to negative values upon PAA5 addition and was subsequently stabilized when the adsorbed PAA amount exceeded the saturation point. Figure S1 indicates that the optimized amount of PAA5 was 1 wt.% of $MgAl_2O_4$. The influence of milling time on particle size is shown in Figure S2 b, and reveals a remarkable decay in the particle size of spinel agglomerates with a longer milling time.

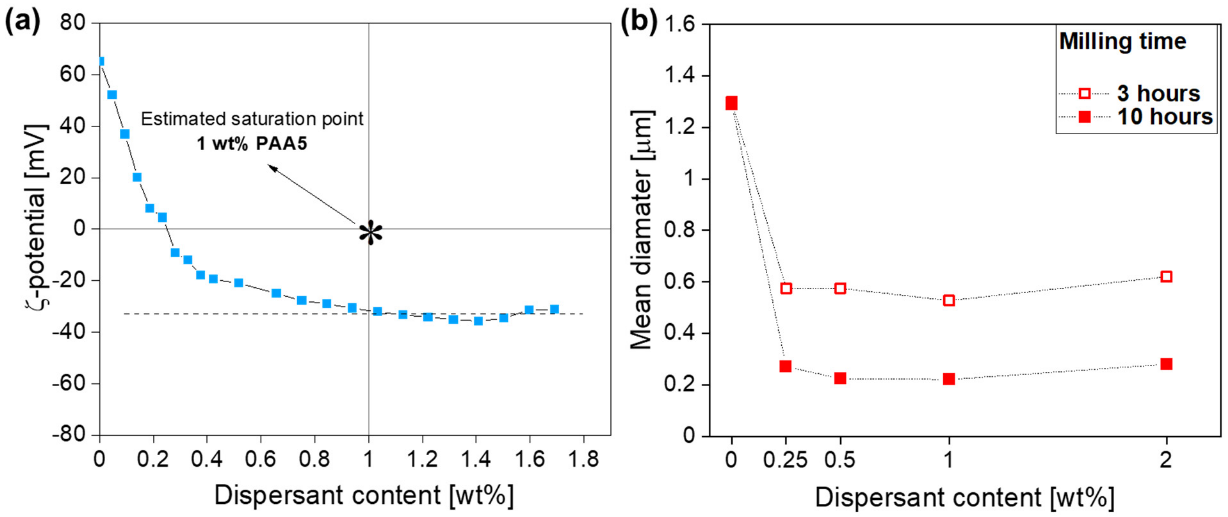

Figure S2. (**a**) Surface charge and (**b**) mean diameter obtained by low-energy ball-milling after 3 and 10 hours of $MgAl_2O_4$ as a function of PAA5 dispersant quantity.

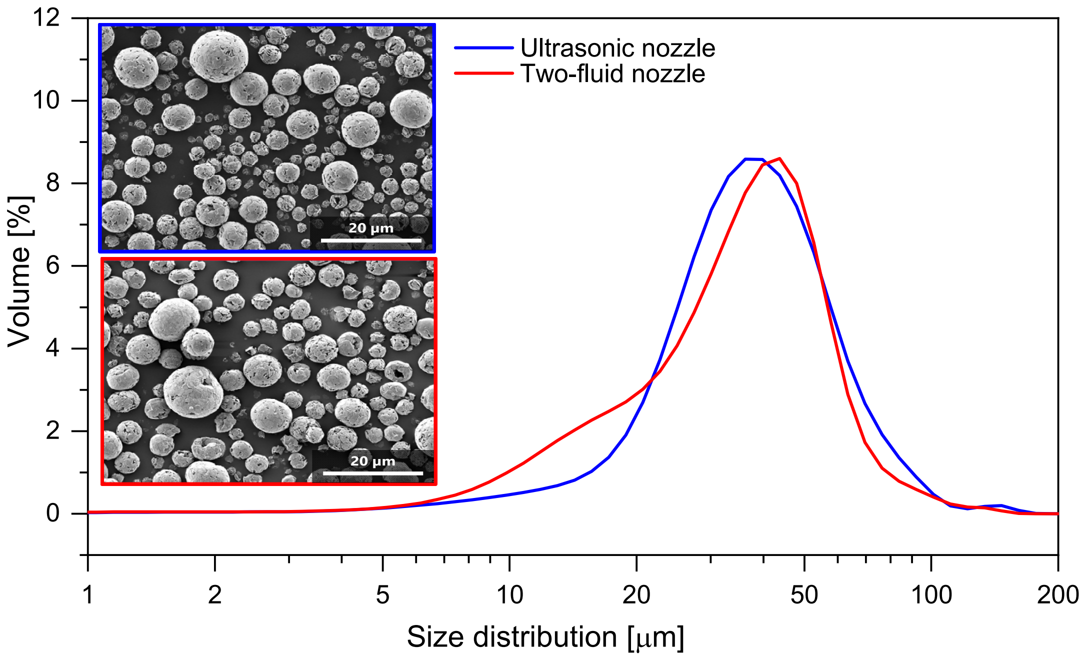

Figure S3. Particle size distribution and morphology of MgAl granules spray-dried using an ultrasonic two-fluid nozzle.

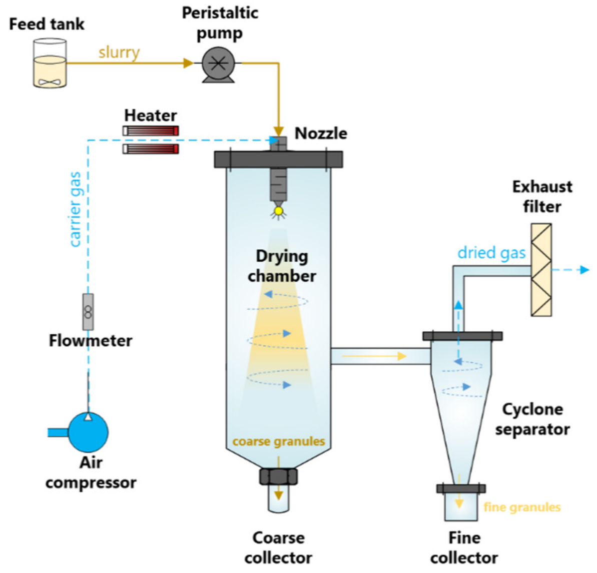

Figure S4. Schematic representation of the spray-drying process.

Details of characterization methods. The crystalline phases of the materials were characterized by X-ray diffraction (XRD) using the PANalytical X'Pert PROh–2h (PANalytical, Almelo, Netherlands) scan system equipped with a Johansson monochromator (Cu Kα1 radiation, 1.5406 Å) and an X'Celerator linear detector. The scanning range, step interval, and time per step were fixed as 5–80 °, 0.017°, and 99.7 s, respectively.

In order to determine the surface area of the materials, N_2 adsorption and desorption isotherms were measured at 77 K in the SA 3100 Surface Area Analyzer (Beckman Coulter, Krefeld, Germany). Materials were dried at 180 °C for 2 h in synthetic air, using the SA-PREP Surface Area Outgasser (Beckman Coulter, Krefeld, Germany), and the surface area was calculated using the Brunauer, Emmett, and Teller (BET) [3] model.

Surface and sub-surface (i.e., cross-section) morphology of the materials were evaluated by scanning electron microscopy (SEM) (Tescan SEM VEGA3, Tescan instruments, Brno, Czech Republic). For cross-sectional imaging, granules were initially mounted in epoxy resin and subsequently ground and polished with a Struers Tegramin-30 polisher (Struers GmbH, Willich, Germany). Multi-step polishing (1 to 40 μm) was applied using water-based diamond suspensions and hard composite discs for the removal of material from the surface until the sub-surface was reached. Energy-dispersive X-ray spectroscopy (EDX) analysis was performed to evaluate the elemental mapping using Bruker's XFlash 6 detector (Bruker Nano GmbH, Berlin, Germany).

Surface charge of the materials was determined by the Zeta Probe Analyzer from Colloidal Dynamics based on an electroacoustic method (Colloidal Dynamics, LLC, Palm Valley, Florida, USA). All suspensions with 5 wt.% of solid loading were titrated at room temperature in the range pH 3 to 13 using 0.1M HCl and 0.1M NaOH solutions. The pH step was set as 0.5 and the equilibrium time was 30 seconds, with a magnetic stirrer velocity equal to 380 rpm.

Particle size distribution of the raw powders and granules was screened using the LS 13 320 Laser Diffraction Particle Size Analyzer from Beckman Coulter, Germany, in a wet module. Helium pycnometry (AccuPyc II 1340, Micromeritics Inc, Norcross, Georgia, USA) was used to measure the absolute densities of the powders.

Mercury intrusion porosimeter tests were conducted to determine the total porosity and pore size distribution of the materials (Pascal 140/440, Thermo Fisher, Germany). The surface tension and contact angle of mercury were set as 0.48 N/m and 140°, respectively. For each measurement, ~150 mg of calcined granules was used. For low-pressure measurement, two intrusion cycles were performed up to 400 kPa, respectively. To access smaller pores (<5 µm), a high-pressure measurement was performed up to 200 MPa in Pascal 440.

Enumeration of the phages. For enumeration of phages, the double agar layer (DAL) method was applied. To increase the attachment of the phages to the bacterial hosts [4], 5 mM of $MgSO_4$ (in MS2-relevant tests) or $CaCl_2$ (in fr-relevant tests) was added to TRIS buffer (0.02 M tris(hydroxymethyl)-aminomethane (Merck), pH = 7.3), soft agar (32 g Tryptic Soy Agar (TSA, VWR Chemicals, Radnor, Pennsylvania, USA) in 1 L), and hard agar solutions (40 g TSA in 1 L). Each solution was autoclaved prior to filtration tests. In a typical plating, 1 mL of phage-containing sample was mixed with 200 µL of *E. coli* suspension in 2.5 mL of soft agar that was previously kept at 55 °C, and was poured onto a plate containing ca. 15 mL of hard agar and incubated overnight at 37 °C for plaque formation and counting. Duplicate plating was performed for each dilution.

Filtration tests. Virus retention tests were performed in a laboratory-scale filtration set-up, where 4 g of filter material was placed over glass fiber filter paper (pore size 0.4 µm, binder-free, Macherey-Nagel filters) in a 70 mm-long cartridge with a diameter of 15 mm. To ensure maximal packing density, stabilize flow rates, and prevent preferential water flow pathways due to the presence of air between granules, cartridges were tapped 1000 times using a jolting volumeter (JEL STAV II, J. Engelsmann AG, Ludwigshafen, Germany) and subsequently gently mixed together with DI water shortly before the filtration procedure to ensure there was no trapping of air bubbles.

The initial solution containing 10^7 PFU·mL^{-1} of appropriate virus surrogates in TRIS buffer was prepared. TRIS buffer with a pKa of 8.1 is an effective buffer between pH 7 and 9 and was selected in order to maintain a stable pH during the filtration process, used for diluting the solutions [5]. The pH of the solution was fixed to 7.3 using HCl. Two liters of virus solution was passed through each cartridge using a peristaltic pump (IPC High-Precision Multichannel Dispenser, Ismatec, Zurich, Switzerland) at a flow rate of 5 mL·min^{-1}.

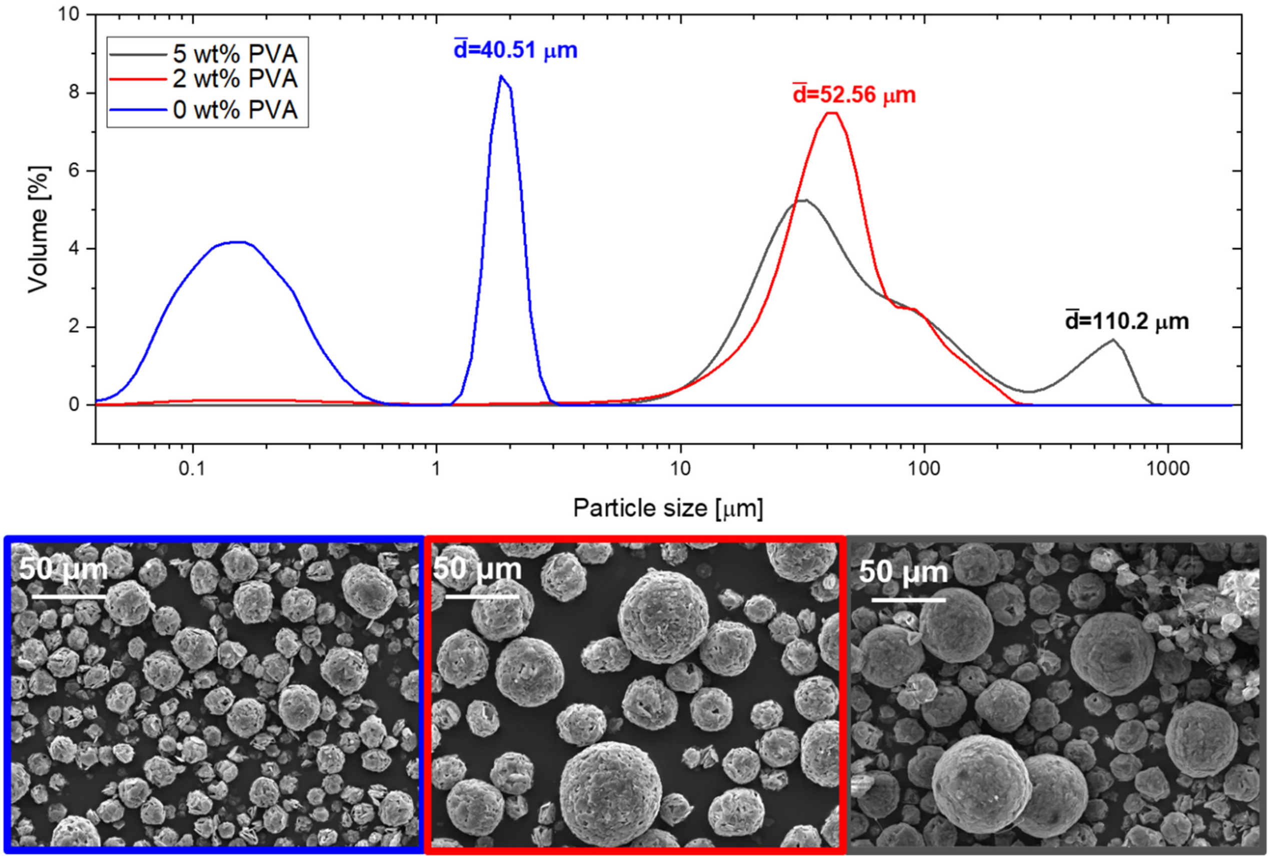

Figure S5. Particle size distribution and surface morphology of MgAl granules based on different PVA binder quantities.

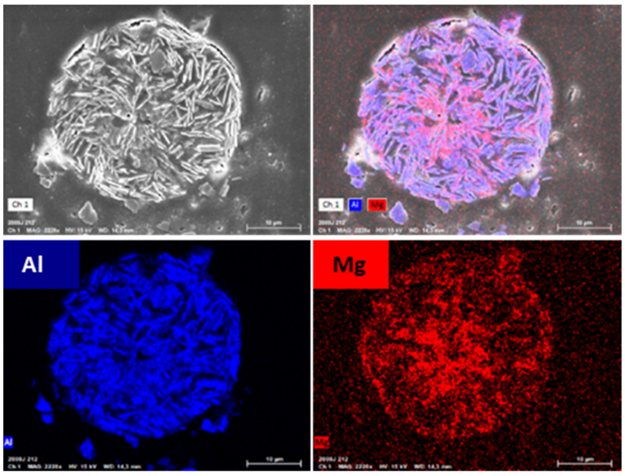

Figure S6. Sub-surface morphology and elemental mapping of MgAl granules.

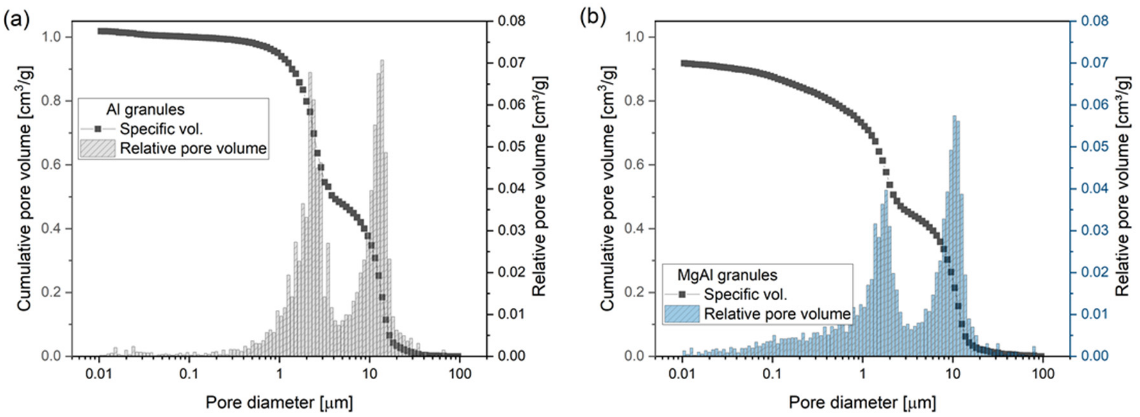

Figure S7. Pore size distribution and cumulative pore volume of the calcined (**a**) Al and (**b**) MgAl granules.

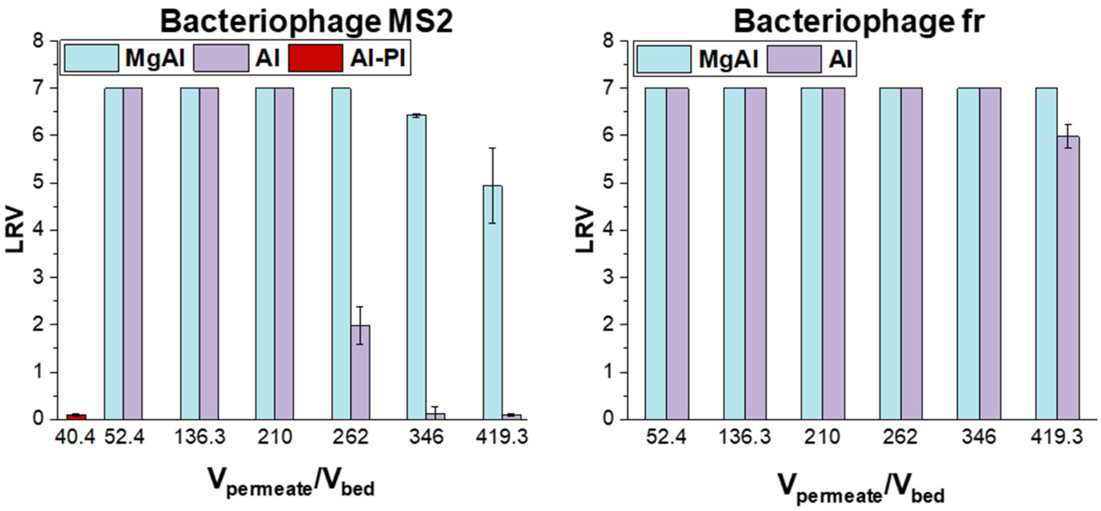

Figure S8. Retention performance of the granules based on MS2 and fr log_{10} removal as a function of normalized filtrate volume per filtered bed volume .

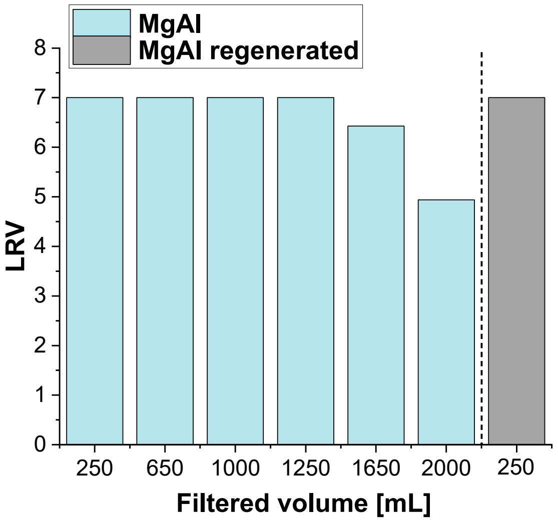

Figure S9. Retention performance of the regenerated MgAl granules at 400 °C in air.

References

1. Asakura, S. and F. Oosawa, *Interaction between particles suspended in solutions of macromolecules.* Journal of polymer science, 1958. **33**(126): p. 183-192.
2. Lu, K. and C. Kessler, *Colloidal dispersion and rheology study of nanoparticles.* Journal of materials science, 2006. **41**(17): p. 5613-5618.
3. Brunauer, S., P.H. Emmett, and E. Teller, *Adsorption of gases in multimolecular layers.* Journal of the American chemical society, 1938. **60**(2): p. 309-319.
4. Carvalho, C., et al., *Method for bacteriophage isolation against target Campylobacter strains.* Letters in applied microbiology, 2010. **50**(2): p. 192-197.
5. Chu, Y., Y. Jin, and M.V. Yates, Virus transport through saturated sand columns as affected by different buffer solutions. 2000, Wiley Online Library.
